# iNKT Cell Production of GM-CSF Controls *Mycobacterium tuberculosis*

**DOI:** 10.1371/journal.ppat.1003805

**Published:** 2014-01-02

**Authors:** Alissa C. Rothchild, Pushpa Jayaraman, Cláudio Nunes-Alves, Samuel M. Behar

**Affiliations:** 1 Program in Immunology, Division of Medical Sciences, Harvard Medical School, Boston, Massachusetts, United States of America; 2 Division of Rheumatology, Immunology, and Allergy, Brigham and Women's Hospital and Harvard Medical School, Boston, Massachusetts, United States of America; 3 Department of Microbiology and Physiological Systems, University of Massachusetts Medical School, Worcester, Massachusetts, United States of America; 4 Life and Health Sciences Research Institute (ICVS), School of Health Sciences, University of Minho, Braga, Portugal; 5 ICVS/3B's-PT Government Associate Laboratory, Braga, Guimarães, Portugal; Portland VA Medical Center, Oregon Health and Science University, United States of America

## Abstract

Invariant natural killer T (iNKT) cells are activated during infection, but how they limit microbial growth is unknown in most cases. We investigated how iNKT cells suppress intracellular *Mycobacterium tuberculosis* (Mtb) replication. When co-cultured with infected macrophages, iNKT cell activation, as measured by CD25 upregulation and IFNγ production, was primarily driven by IL-12 and IL-18. In contrast, iNKT cell control of Mtb growth was CD1d-dependent, and did not require IL-12, IL-18, or IFNγ. This demonstrated that conventional activation markers did not correlate with iNKT cell effector function during Mtb infection. iNKT cell control of Mtb replication was also independent of TNF and cell-mediated cytotoxicity. By dissociating cytokine-driven activation and CD1d-restricted effector function, we uncovered a novel mediator of iNKT cell antimicrobial activity: GM-CSF. iNKT cells produced GM-CSF in vitro and in vivo in a CD1d-dependent manner during Mtb infection, and GM-CSF was both necessary and sufficient to control Mtb growth. Here, we have identified GM-CSF production as a novel iNKT cell antimicrobial effector function and uncovered a potential role for GM-CSF in T cell immunity against Mtb.

## Author Summary

*Mycobacterium tuberculosis* (Mtb) is the cause of tuberculosis, a leading cause of sickness and death worldwide. Although much is known about CD4^+^ and CD8^+^ T cell responses to Mtb, the role of other T cell subsets is poorly understood. Invariant natural killer T (iNKT) cells are innate lymphocytes that express a semi-invariant T cell receptor and recognize lipid antigens presented by CD1d. Although iNKT cells participate in the immune response to many different pathogens, little is known about how iNKT cells directly kill microbes. We previously showed that when co-cultured with Mtb-infected macrophages, iNKT cells inhibit intracellular Mtb replication. Now, we used this model to dissociate the signals that induce iNKT cell activation markers including IFNγ production, from the signals that activate iNKT cell antimicrobial activity. This allowed us to uncover a novel antimicrobial effector function produced by iNKT cells: GM-CSF. GM-CSF is essential for immunity to Mtb, but its role has never been defined. This study is the first report to demonstrate a protective function of GM-CSF production by any T cell subset during Mtb infection. T cell production of GM-CSF should be considered as a potential mechanism of antimicrobial immunity.

## Introduction

CD1 restricted T cells were first proposed to have a role in antimicrobial immunity based on the observations that CD4^−^CD8^−^ (DN) T cells restricted by group 1 CD1 (CD1a, CD1b, and CD1c) recognized unique and complex lipids from the Mtb cell wall [1], [2]. Similarly, invariant natural killer T (iNKT) cell antimicrobial function was originally based on the recognition of microbial lipid or glycolipid molecules presented by the MHC-like molecule CD1d. iNKT cells are now recognized to influence many different immunological conditions including autoimmune disease, asthma and allergy, anti-tumor response, graft-versus-host disease, and infection [3].

There are several pathways by which iNKT cells can be activated. Classically, high affinity antigens that are potent agonists, typified by the synthetic lipid α-galactosylceramide (αGalCer), trigger TCR activation in a CD1d-dependent manner. Several infectious agents produce microbial lipids that are presented on CD1d and recognized by iNKT cells including *Borrelia burgdorferi* and *Sphingomonas capsulata* [4], [5], [6]. Activation can also occur when iNKT cells recognize a weak lower affinity self or microbial ligand, insufficient by itself to induce activation, in the context of costimulatory signals. This mode of activation has been shown for pathogens, such as *Salmonella typhimurium*, viruses such as *Influenza A*, and fungi such as *Aspergillus fumigatus* [7], [8], [9]. There is strong evidence that a major driver of this type of iNKT cell activation is IL-12, which is produced when microbial danger signals stimulate pattern recognition receptors such as Toll-like receptors (TLRs) or dectin-1 [7], [9], [10].

One of the remaining unanswered questions in iNKT cell biology is what specific role these cells have during infection and whether their activation by different pathways leads to the expression of diverse functions. To address the role of iNKT cells during infection, many of these studies have used mouse models that lack iNKT cells (Jα18^−/−^, CD1d^−/−^) or administered αGalCer, a potent activator of iNKT cells. While this strategy has been useful for determining whether iNKT cells are required for host resistance, and for revealing potential antimicrobial effector functions induced after strong activation, much less is known about the physiological role of iNKT cells during infection. Tracking iNKT cell function in vivo has relied extensively on CD69 upregulation and IFNγ production. Interestingly, in the absence of exogenous αGalCer treatment, there is little evidence that IFNγ production by iNKT cells is protective during infection [11]. Given that iNKT cells are capable of producing a variety of different cytokines and chemokines including IL-4, IL-10, IL-17, TNF, GM-CSF, MIP-1α, MIP-1β as well as having immunomodulatory effects via expression of CD40 and other costimulatory ligands [12], it is surprising that it is still unknown for most infections whether iNKT cells have a direct antimicrobial role. Only in select cases have protective mechanisms been defined. For example, iNKT cells are important for neutrophil recruitment to the lung during *Pseudomonas* infection [13] and IFNγ is required for protection by iNKT cells against *Streptococcus pneumoniae*, although this could represent a direct or indirect effect [11].

There are several lines of evidence that activated iNKT cells enhance host resistance to Mtb. Administration of αGalCer, which activates iNKT cells in vivo, significantly prolongs the survival of susceptible mouse strains following Mtb infection and this effect is synergistic with antibiotics [14], [15]. αGalCer activates human iNKT cells to lyse Mtb-infected macrophages and kill intracellular bacteria [16]. Even BCG vaccination is more effective when it is conjugated with αGalCer [17]. Although αGalCer is used as a pharmacological activator of iNKT cells in this context, it is not required. iNKT cells cultured with Mtb-infected primary macrophages stimulate antimicrobial activity that restricts bacterial growth and adoptive transfer of iNKT cells limits bacterial growth in vivo [18]. Finally, several clinical studies find that a decrease of iNKT cells in the periphery is a marker of active disease compared to latent infection or healthy controls [19], [20], [21]. Despite these findings of iNKT cell activation leading to enhanced control, iNKT cells are dispensable in the murine model of chronic tuberculosis infection [22], [23], [24], [25].

Our model, in which iNKT cells activate infected macrophages to control Mtb infection in the absence of exogenous stimulation (e.g., αGalCer), provided a unique opportunity to define the direct effector functions of iNKT cells. This model allowed us to study the interaction between iNKT cells and Mtb-infected macrophages and provided the opportunity to perturb specific signaling and effector pathways and then measure subsequent changes in bacterial control. Greater insight into the effector function of iNKT cells during Mtb infection could lead to novel therapeutic approaches for augmenting their antimicrobial capacity and boosting the host immune response during infection [14], [15].

Here we report that iNKT cells upregulated activation markers and produced IFNγ during Mtb infection in vitro and these markers of activation were driven largely by IL-12 and IL-18. Blocking these cytokine signals almost completely inhibited IFNγ production, but surprisingly had little effect on the ability of iNKT cells to control Mtb growth. In contrast, the antimicrobial function of iNKT cells required CD1d signaling and was mediated by a soluble factor, GM-CSF. Blocking GM-CSF abrogated restriction of bacterial growth by iNKT cells and GM-CSF was sufficient to inhibit mycobacterial growth in vitro. We identified GM-CSF as an antimycobacterial effector molecule produced by iNKT cells with the ability to suppress Mtb infection.

## Results

### iNKT cells are activated by Mtb-infected macrophages

**Figure 1:**
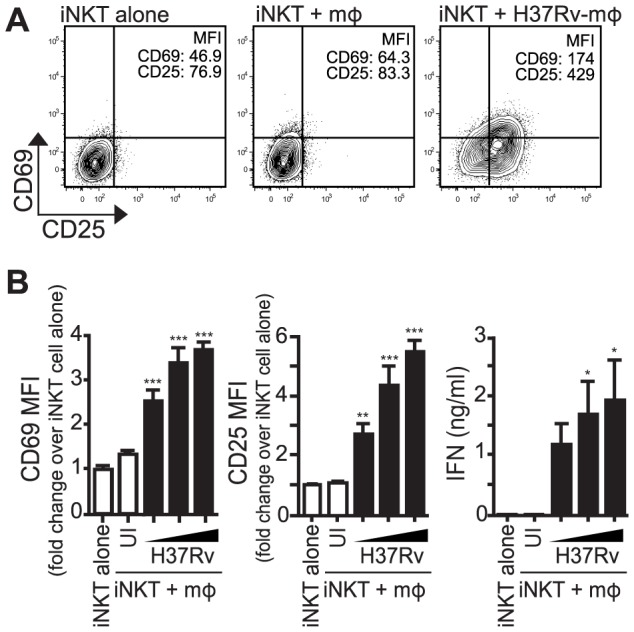
iNKT cells are activated by Mtb-infected mϕ. (A) iNKT cells were cultured either alone, with uninfected thioglycollate-elicited peritoneal mϕ, or H37Rv-infected mϕ for 24 hours. Cells were stained for CD69 and CD25 and mϕ were distinguished from iNKT cells by F4/80 staining. (B) Fold change in CD69 and CD25 MFI on iNKT cells cultured with uninfected or H37Rv-infected mϕ compared to iNKT cells alone. Supernatant was harvested at 24 hours and IFNγ measured by ELISA. Error bars indicate mean ± SEM. *P<.05, **P<.01, ***P<.001. (One-way ANOVA with Dunnet's post-test, compared to iNKT cells alone). Data are representative of eight independent experiments. Mϕ, macrophage; UI, uninfected; 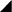 , MOI titration, 1.5∶1, 3∶1, 6∶1.

CD69 and CD25, both classic T cell activation markers, as well as IFNγ production, were chosen to track the activation of iNKT cells by Mtb-infected macrophages. Primary mouse iNKT cell lines were co-cultured with thioglycollate-elicited peritoneal macrophages infected with increasing multiplicity of infection (MOI) of H37Rv, a virulent strain of Mtb. After 24 hours, iNKT cells cultured with Mtb-infected macrophages expressed higher levels of CD69 and CD25 and produced more IFNγ compared to iNKT cells cultured with uninfected macrophages (Figures 1A and 1B). A similar activation pattern was observed when hepatic mononuclear cells (HMNC), a source of primary uncultured iNKT cells, were used (Figure S1A) or when iNKT cells were cultured with H37Rv-infected bone marrow derived macrophages (BMDM) (Figure S1B). These data indicate that iNKT cells become activated after stimulation with Mtb-infected APCs.

### iNKT cell activation by Mtb-infected macrophages requires a combination of cytokine and CD1d-dependent signals

**Figure 2:**
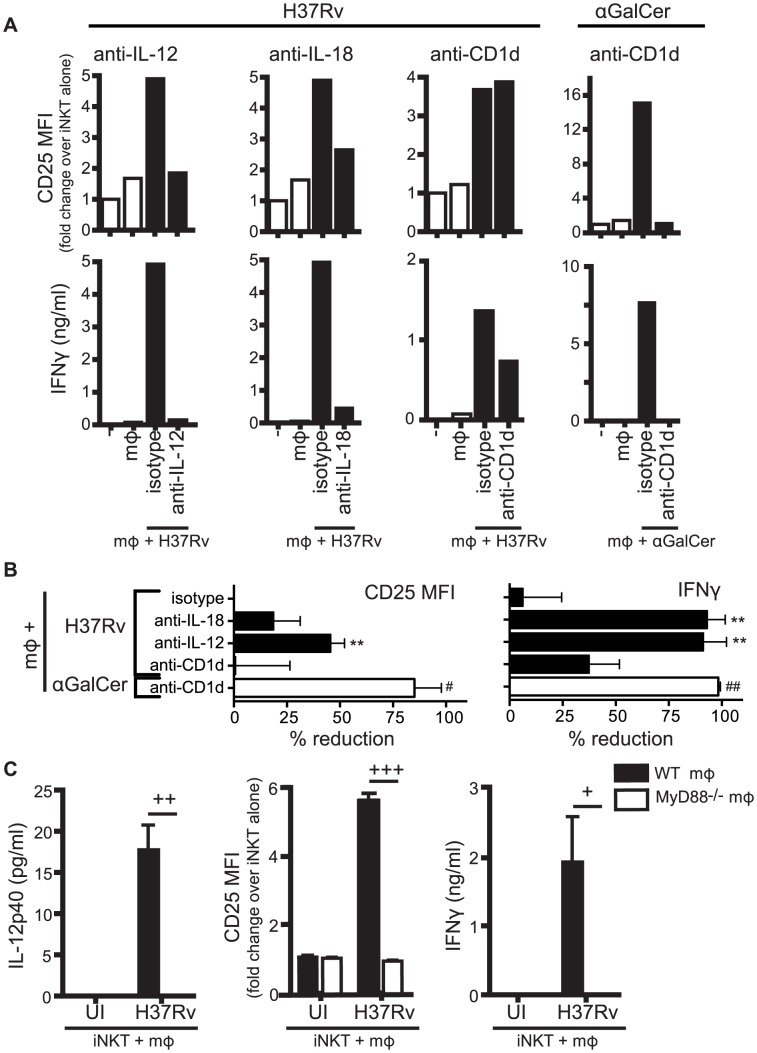
Production of IL-12 and IL-18 by Mtb-infected mϕ induce traditional markers of iNKT cell activation. (A) iNKT cells were cultured either alone, with uninfected mϕ, H37Rv-infected mϕ, or αGalCer-loaded mϕ for 24 hours in the presence of blocking antibodies against IL-12p40 (20 µg/ml), IL-18 (10 µg/ml), CD1d (20 µg/ml), or respective isotype controls. Cells were stained for CD25 and mϕ were distinguished from iNKT cells by F4/80 staining. Supernatant was harvested at 24 hours and IFNγ measured by ELISA. (B) % reduction calculated as 100*[(iNKT_H37Rv-mϕ_−iNKT_alone_)−(iNKT_Ab+H37Rv-mϕ_−iNKT_alone_)]/(iNKT_H37Rv-mϕ_−iNKT_alone_). Conditions with αGalCer stimulation calculated similarly. (C) iNKT cells were cultured with uninfected or H37Rv-infected WT or MyD88^−/−^ mϕ for 24 hours. Cells were stained for CD25 and mϕ were distinguished from iNKT cells by F4/80 staining. Supernatant was harvested at 24 hours and IFNγ and IL-12p40 measured by ELISA. Error bars indicate mean ± SEM. **P<0.01 compared to isotype control. (One-way ANOVA with Dunnet's post-test). #P<0.05, ##P<0.01 compared to isotype control (data not shown) (unpaired Student's t-test). +P<0.05, ++P<0.01, +++P<0.001 (unpaired Student's t-test). Data are representative of, or compiled from, (A,B) three (anti-CD1d), four (anti-IL-12), two (anti-IL-18) and (C) two independent experiments.

Infection by various microbes including *Salmonella*, *Aspergillus*, and *E. coli* LPS, induce iNKT cell activation by a combination of IL-12, IL-18, and/or TCR stimulation through interaction with CD1d [7], [9], [10], [26]. To determine whether these signals were required for the activation of iNKT cells by Mtb-infected macrophages, we added neutralizing antibodies to cell co-cultures and measured iNKT cell activation after 24 hours. We found that CD25 and IFNγ were inhibited to varying degrees by blockade of the activating signals (Figures 2A and 2B). CD69 expression was more variable and blocking antibodies had little effect on its expression (data not shown). Blocking cytokine signals (IL-12p40, IL-18) had a greater inhibitory effect on the markers than blocking the TCR-CD1d interaction. For example, anti-IL-12p40 reduced CD25 surface expression by 45.7±3.3%, and inhibited IFNγ production nearly completely (91.4±4.4%) (mean ± SEM, n = 4 experiments) (Figures 2A and 2B). In contrast, anti-CD1d had no effect on CD25 expression and only a modest effect on IFNγ production (37.5±6.4% reduction) (mean ± SEM, n = 3–4 experiments). The failure of anti-CD1d to block iNKT cell activation was not due to a problem with the experimental conditions since anti-CD1d blocked induction of CD25 and abrogated IFNγ production after α-GalCer stimulation of iNKT cells (Figures 2A and 2B). To verify that cytokines were driving iNKT cell activation, iNKT cells were cultured with MyD88^−/−^ macrophages, which do not produce IL-12 after H37Rv infection [27] (Figure 2C). When co-cultured with Mtb-infected MyD88^−/−^ macrophages, iNKT cells did not upregulate CD25 or secrete IFNγ. These results indicated that IL-12 and IL-18 produced by macrophages during Mtb infection drove the activation of iNKT cells as measured by induction of CD25 surface expression and IFNγ production. Thus, these conventional markers of iNKT cell activation were not reliable indicators of CD1d-dependent TCR-mediated signaling during Mtb infection.

### Control of intracellular Mtb growth by iNKT cells requires CD1d but not IL-12 and IL-18

**Figure 3:**
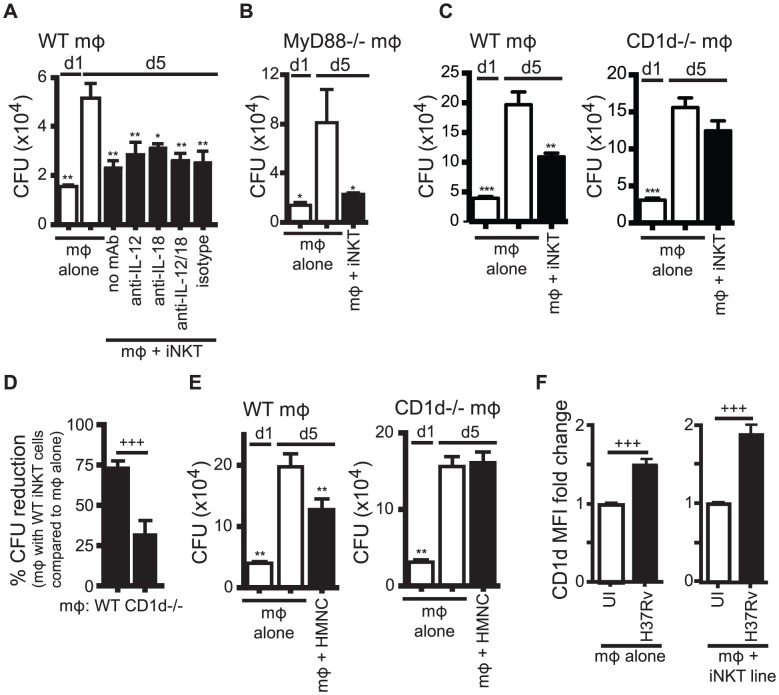
iNKT cell mediated control is CD1d-dependent but does not require IL-12 or IL-18. (A) Colony forming unit (CFU) assay measuring Mtb bacterial growth in H37Rv-infected WT mϕ on d1 and d5 post-infection. iNKT cells, anti-IL-12p40, anti-IL-18 blocking or isotype control antibodies were added on d1 after infection. (B) CFU assay d1 and d5 post-infection for H37Rv-infected MyD88^−/−^ mϕ with iNKT cells added on d1. (C, E) CFU assay d1 and d5 post-infection for H37Rv-infected WT and CD1d^−/−^ mϕ with iNKT cells added on d1 at a ratio of 1∶1 (C) or HMNC at a ratio of 3∶1 (E). (D) Compiled data from 6 independent experiments as described in (C). (F) H37Rv-infected mϕ after 24 hours. CD1d MFI fold change over uninfected mϕ either without or with iNKT cells. Error bars indicate mean ± SEM. *P<0.05, **P<0.01, ***P<0.001 (One-way ANOVA with Dunnet's post-test, compared to d5 untreated mϕ). +++P<.001 (unpaired Student's t-test). Data are representative of two (A, B) six (C, D), and one (E) independent experiment(s) with three or more replicates, or more than 12 independent experiments (F).

As shown previously, iNKT cells cultured with H37Rv-infected macrophages reduced intracellular bacterial growth over a 5 day infection [18] (Figure 3A). We used this co-culture model to evaluate how the different activation signals affected iNKT cell antimicrobial activity.

We first tested the requirement for IL-12 and IL-18 signaling. The addition of anti-IL-12p40 and/or anti-IL-18 blocking antibodies to co-cultures of iNKT cells and H37Rv-infected WT macrophages did not affect the ability of iNKT cells to inhibit bacterial growth (Figure 3A). We confirmed this result using MyD88^−/−^ macrophages, which did not induce upregulation of CD25 or IFNγ by iNKT cells (Figure 2C). iNKT cells were able to inhibit bacterial growth in H37Rv-infected MyD88^−/−^ macrophages (Figure 3B). These findings were unexpected because they suggested that the traditional markers of activation did not correctly predict iNKT cell antimycobacterial function.

We next addressed whether CD1d expression by the infected macrophages was required. iNKT cells were able to limit intracellular bacterial growth only if the infected macrophages expressed CD1d (Figure 3C). Normalizing the bacterial growth in each experiment allowed us to determine the requirement for CD1d across multiple experiments (see Experimental Procedures for details). When Mtb growth inhibition by iNKT cells in WT and CD1d^−/−^ macrophages were compared in this manner, iNKT cells suppressed growth of Mtb in WT macrophages significantly more than in CD1d^−/−^ macrophages (73.1±4.7% CFU reduction vs. 31.5±9.3%) (mean ± SEM, n = 6 experiments, p = 0.0002) (Figure 3D). To confirm the dependence on CD1d for bacterial control by iNKT cells, and to show that the culture conditions of the primary cell lines had not significantly altered the effector functions of the iNKT cells, we repeated this experiment using HMNC as a source of primary uncultured iNKT cells. We found that HMNCs also required CD1d signaling for inhibition of bacterial growth (Figure 3E).

Since CD1d expression by macrophages was required to elicit optimal iNKT cell effector function, we considered whether Mtb infection altered the CD1d surface expression on macrophages. After 24 hours, H37Rv infection led to a modest increase in CD1d surface expression on macrophages (1.5±0.1 fold change) (mean ± SEM, n = 13 experiments, p<0.0001) (Figure 3F). Co-culture with iNKT cells led to a slight increase in CD1d expression during H37Rv infection (1.9±0.1 fold change) (mean ± SEM, n = 22 experiments, p<0.0001) (Figure 3F). These increases were averages for the bulk culture and may have underestimated the increase for individually infected macrophages, because not every macrophage was infected.

These results indicated that the cytokines (IL-12, IL-18) that drive expression of CD25 and IFNγ production were not required to stimulate iNKT cell antimycobacterial effector functions. In contrast, CD1d expression, which played only a small role in stimulating IFNγ production, was required for iNKT cell mediated control of intracellular Mtb growth. The dissociation between standard measurements of iNKT cell activation from iNKT cell effector function in response to Mtb infection was unexpected.

### iNKT cells exhibit an antimycobacterial function independent of IFNγ

**Figure 4:**
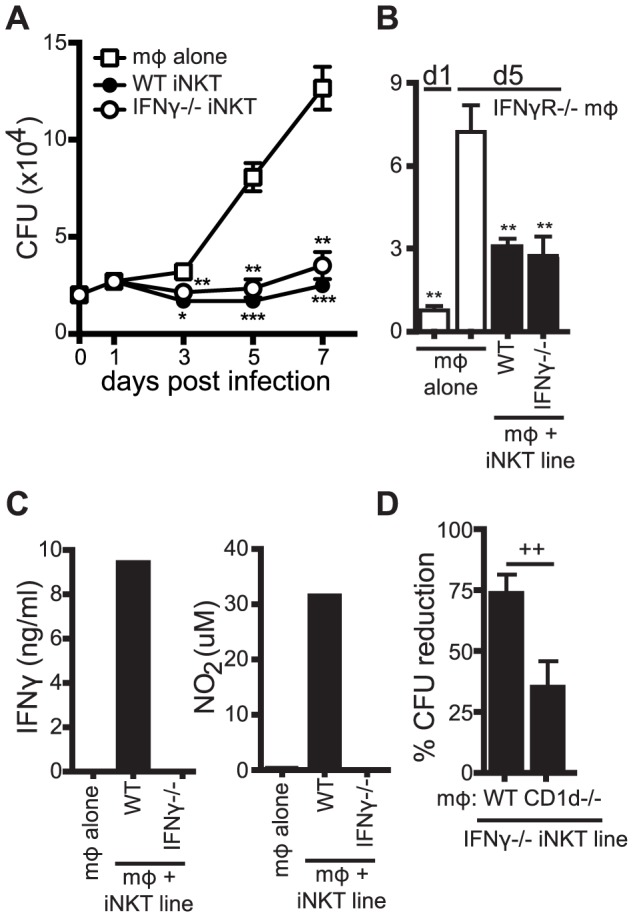
iNKT cell mediated control is independent of IFNγ. (A) CFU for H37Rv-infected WT mϕ with WT and IFNγ^−/−^ iNKT cells added on d1 post-infection. CFU were measured on d0 (mϕ alone) and on d1, d3, d5 and d7 post-infection. (B) CFU assay d1 and d5 post-infection for H37Rv-infected IFNγR^−/−^ mϕ with WT and IFNγ^−/−^ iNKT cells added on d1. (C) IFNγ and nitrite, a stable breakdown product of NO_2_, measured for H37Rv-infected WT mϕ with WT and IFNγ^−/−^ iNKT cells added on d1 post-infection. (D) Compiled data from 5 independent experiments of the CFU assay d1 and d5 post-infection with H37Rv-infected WT and CD1d^−/−^ mϕ with IFNγ^−/−^ iNKT cells added on d1. Error bars indicate mean ± SEM. *P<0.05, **P<0.01, ***P<.001 (One-way ANOVA with Dunnet's post-test, compared to d3, d5, or d7 untreated mϕ). ++P<.01 (Unpaired Student's t-test). Data are representative of or cumulative from 13 (d5) and four (d7) (A), two (B) three (C), or four (D) independent experiments with three or more replicates.

Since IL-12 and IL-18 blockade largely inhibited IFNγ production but had little impact on CFU control, we considered whether iNKT cell antimicrobial effector function was independent of IFNγ. To test this, we derived an IFNγ^−/−^ iNKT cell line. When IFNγ^−/−^ iNKT cells were added to H37Rv-infected macrophages, they inhibited bacterial growth similar to WT iNKT cells (Figure 4A). Over multiple experiments, WT and IFNγ^−/−^ iNKT cells reduced bacteria growth similarly by d5 by 60.0±4.7% versus 60.1±4.0%, respectively (mean ± SEM, n = 13 experiments, p = NS) (Figure S2). A similar result was found by d7 post-infection (Figure S2).

IFNγ plays an important antimicrobial role during Mtb infection. To be certain that iNKT cells were controlling bacterial growth independently of IFNγ signaling, WT and IFNγ^−/−^ iNKT cells were cultured with H37Rv-infected IFNγR^−/−^ macrophages. The iNKT cells still limited bacterial growth by d5 in these cells (Figure 4B). We also examined whether iNKT cells controlled Mtb growth through nitric oxide, an important mediator of antimycobacterial immunity produced during infection by IFNγ stimulation of the enzyme inducible nitric oxide synthase (iNOS) [28]. Neither IFNγ nor nitrite, a stable breakdown product of nitric oxide, were detected after addition of IFNγ^−/−^ iNKT cells, while both were detected when WT iNKT cells were present (Figure 4C). Furthermore, naïve splenocytes (as a source of iNKT cells [18]) inhibited bacterial replication similarly in WT, IFNγR^−/−^, and iNOS−/− macrophages (data not shown). We next tested whether the IFNγ^−/−^ iNKT cells also required CD1d to mediate their antimicrobial effector function. Similar to WT iNKT cells, IFNγ^−/−^ iNKT cells inhibited growth of Mtb in WT macrophages significantly more than in CD1d^−/−^ macrophages by 73.3±7.2% versus 34.4±10.6% (mean ± SEM, n = 5 experiments, p = 0.0043) (Figure 4D). These data demonstrated that iNKT cells were capable of controlling Mtb growth independently of IFNγ and nitric oxide and that the IFNγ-independent effector function required CD1d-mediated activation.

### iNKT cell antimicrobial function is independent of cytolytic activity and TNF

**Figure 5:**
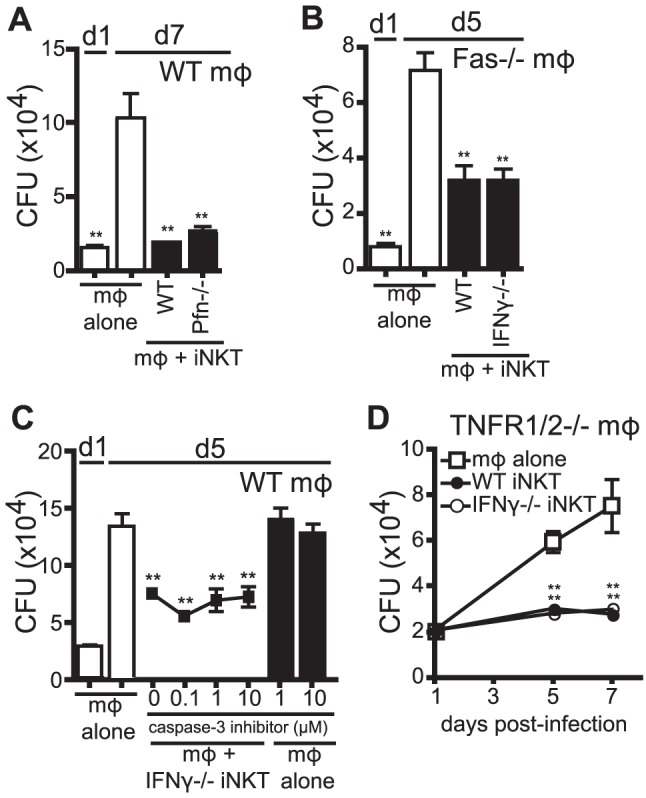
IFNγ-independent antimicrobial effector function of iNKT cells is independent of cytolytic activity. (A–D) CFU assay d1, d5, and/or d7 post-infection with H37Rv-infected WT mϕ (A, C), Fas^−/−^ mϕ (B), or TNFR1/2^−/−^ mϕ (D) with WT iNKT cells (A, B, D), IFNγ^−/−^ iNKT cells (B–D), or Prf^−/−^ iNKT cells (A) added on d1 post infection at a 1∶1 ratio. (C) H37Rv-infected mϕ were treated with 0.1–10 µM of caspase-3 inhibitor peptide (Z-DEVD-FMK) 2 hours prior to addition of iNKT cells. Error bars indicate mean ± SEM. *P<0.05, **P<0.01 (One-way ANOVA with Dunnet's post-test, compared to d5 or d7 untreated mϕ.) Data are representative of three (A, C, D) or two (B) independent experiments with three or more replicates.

Human iNKT cells stimulated with αGalCer were shown to control Mtb infection in human monocyte-derived macrophages through granulysin-mediated cytolytic activity [16]. Based on this finding, we determined whether iNKT cells, in the absence of additional stimulation, inhibited Mtb growth through cytolytic activity. To block the major pathways of cytolysis, we added Perforin^−/−^ (Pfn^−/−^) iNKT cells to H37Rv-infected WT macrophages (Figure 5A). In addition, we added either WT or IFNγ^−/−^ iNKT cells to Fas^−/−^ macrophages (Figure 5B). Under these conditions, iNKT cells still significantly suppressed Mtb growth in macrophages. To eliminate the possibility of redundancy between cytolytic pathways, Pfn^−/−^ iNKT cells were added to H37Rv-infected Fas^−/−^ macrophages, and despite elimination of both of the dominant CTL pathways, iNKT cells were still able to significantly inhibit bacterial growth (data not shown). These data show that the major cytolytic pathways were not required for iNKT cell antimicrobial function in this system.

We also took an alternative approach to inhibit cytolytic activity by blocking an important downstream effector of cytolytic activity, caspase-3. Using a specific peptide inhibitor to inhibit caspase-3 activity and apoptosis in target cells, we found that addition of caspase-3 inhibitor to co-cultures of IFNγ^−/−^ iNKT cells and H37Rv-infected macrophages, at concentrations that block apoptosis of infected macrophages [29], did not affect the ability of IFNγ^−/−^ iNKT cells to inhibit bacterial growth (Figure 5C).

TNF plays an important antimicrobial role during Mtb infection [30] and we detected TNF, albeit at low levels, in our co-culture system (data not shown). Therefore, we used TNFR1/2^−/−^ macrophages, which lack both TNF receptors, to test whether TNF signaling was required for iNKT cell control of Mtb. Both WT and IFNγ^−/−^ iNKT cells inhibited bacterial growth when cultured with H37Rv-infected TNFR1/2^−/−^ macrophages, indicating that TNF did not mediate the antimicrobial effector function of IFNγ^−/−^ iNKT cells (Figure 5D). Finally, based on the newly appreciated role of IL-1β in limiting growth of Mtb in macrophages [31], [32], we determined whether IL-1β mediated iNKT cell control of bacterial growth. IFNγ^−/−^ iNKT cells cultured with IL-1R^−/−^ macrophages controlled Mtb growth, ruling out a role for IL-1β signaling (data not shown).

These data showed that iNKT cells controlled Mtb growth independently of cytolytic activity and cytokines previously identified as antimycobacterial, TNF and IL-1β. They suggested that iNKT cells limit bacterial growth through a non-classical effector function.

### iNKT cells secrete a soluble factor with antimicrobial properties

**Figure 6:**
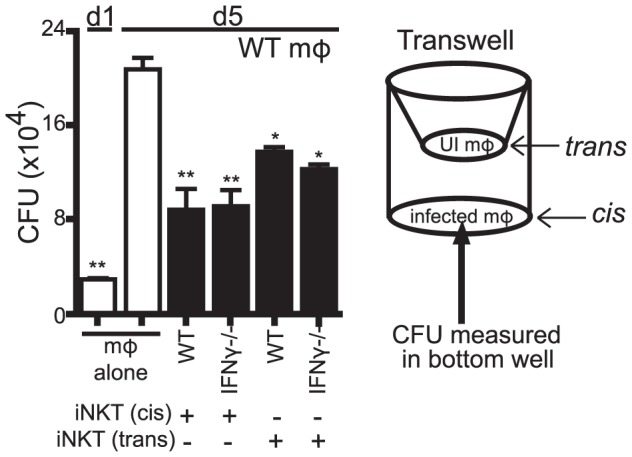
The antimicrobial effector function of iNKT cells is a soluble factor. Transwell CFU assay for H37Rv-infected WT mϕ in a 24-well plate with either WT or IFNγ^−/−^ iNKT cells added directly (cis) or 0.4 µm transwell inserts with WT or IFNγ^−/−^ iNKT cells in the presence of uninfected WT mϕ (trans) added on d1. Error bars indicate mean ± SEM. *P<0.05, **P<0.01 (One-way ANOVA with Dunnet's post-test, compared to d5 untreated mϕ.) Data are representative of two independent experiments with four replicates each.

We next determined whether the antimicrobial activity was a soluble factor or cell contact dependent. Using a transwell system, we found that both WT and IFNγ^−/−^ iNKT cells placed in trans from H37Rv-infected macrophages were able to suppress Mtb growth (Figure 6). Importantly, this effect was only observed if the iNKT cells were in contact with other macrophages in the transwell, but not when the iNKT cells were cultured alone. This was consistent with our observation that induction of the iNKT cell effector function required CD1d signaling via macrophage contact. The finding that contact between iNKT cells and uninfected macrophages was sufficient to induce the antimicrobial activity raised the possibility that a mediator secreted by infected macrophages (e.g., in trans) augmented iNKT cell activation, either by increasing macrophage CD1d expression or by costimulating iNKT cell activation. Most importantly, this experiment confirmed that iNKT cells required cell contact and CD1d expression for their activation, and indicated that their antimicrobial activity was mediated by a soluble factor.

We also found that αGalCer stimulation of IFNγ^−/−^ iNKT cells cultured in trans boosted their ability to control bacterial growth (data not shown). Furthermore, conditioned media produced from IFNγ^−/−^ iNKT cells stimulated with αGalCer-loaded WT but not CD1d^−/−^ macrophages stimulated bacterial control when added to macrophages (Figure S3). We then used conditioned media samples derived from IFNγ^−/−^ iNKT cells stimulated by unloaded or αGalCer-loaded WT or CD1d^−/−^ macrophages for screening purposes. These samples were size fractionated at 10 kDa and 50 kDa MW cutoffs. We identified several cytokines present at high levels only in the fractions that had antimicrobial activity: GM-CSF, TNF, and IL-4 (Figure S3). Since TNF had been previously eliminated and IL-4 is not known to enhance bacterial control [33], we investigated whether iNKT cells produced GM-CSF after co-culture with Mtb-infected macrophages and whether it had antimycobacterial activity.

### iNKT cells produce GM-CSF during Mtb infection in a CD1d-dependent manner and it is critical for controlling Mtb growth

**Figure 7:**
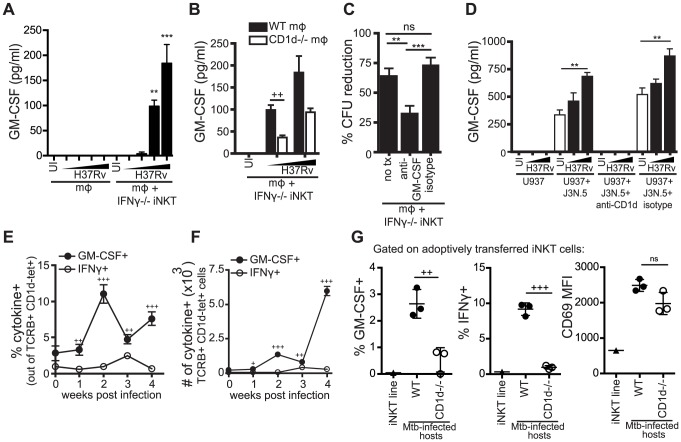
iNKT cells produce GM-CSF during Mtb infection in a CD1d-dependent manner and it is critical for controlling Mtb growth. (A, B) IFNγ^−/−^ iNKT cells added to uninfected or H37Rv-infected WT (A, B) and CD1d^−/−^ mϕ (B). Murine GM-CSF measured in supernatant harvested after 24 hours. (C) % CFU reduction calculated from CFU assays for H37Rv-infected WT mϕ with IFNγ^−/−^ iNKT cells added on d1 in the presence of anti-GM-CSF blocking antibody (10–50 µg/ml) or isotype control. (D) J3N.5 human iNKT cell clone added to uninfected or H37Rv-infected U937 cells in the presence of anti-human-CD1d blocking antibody or isotype control. Human GM-CSF measured in supernatant harvested after 24 hours. (E, F) Lung mononuclear cells from WT Mtb-infected mice were incubated with brefeldin A for four hours at 37°C and then stained. iNKT cells were identified as TCRβ^+^ and CD1d-tetramer^+^. Percentage (E) and number (F) of iNKT cells producing GM-CSF and IFNγ. (G) % GM-CSF^+^, % IFNγ^+^, and CD69 MFI for iNKT cells transferred iv into WT or CD1d^−/−^ Mtb-infected hosts and iNKT cells cultured for 20 hours in basic media. Lung mononuclear cells were treated and stained as in (E, F). Transferred iNKT cells were distinguished from host cells by eFluor 450 staining. Error bars indicate mean ± SEM. +P<.05, ++P<.01, +++P<.001 (unpaired Student's t-test, GM-CSF^+^ versus IFNγ^+^ for respective time points (E, F), WT versus CD1d^−/−^ hosts (B, G)). **P<0.01, ***p<0.001 (One-way ANOVA with Tukey post-test (C), and Dunnet's post-test (A, D), compared to uninfected mϕ). Data are representative of or compiled from four (A, C), two (B), and three (D, G) independent experiments with three or more replicates each or two independent experiments with 5 mice each (E, F). 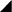 , MOI titration approximately 0.5∶1–3∶1 (A, B) and 2∶1–10∶1 (D).

We detected GM-CSF production 24 hours after co-culture of both WT and IFNγ^−/−^ iNKT cells with H37Rv-infected macrophages (Figure 7A, Figure S4). Because we had already established that the IFNγ-independent antimicrobial function was CD1d-dependent, we tested whether GM-CSF production by iNKT cells required CD1d expression. We found that IFNγ^−/−^ iNKT cells produced significantly more GM-CSF when cultured with Mtb-infected WT macrophages than with Mtb-infected CD1d^−/−^ macrophages; however, some GM-CSF was produced even in the absence of CD1d, especially at higher Mtb MOI (Figure 7B).

To determine if GM-CSF was required for iNKT cell-mediated control in vitro, we added anti-GM-CSF blocking antibodies to the co-culture of IFNγ^−/−^ iNKT cells and Mtb-infected macrophages. GM-CSF blockade impaired bacterial control by IFNγ^−/−^ iNKT cells leading to a significant increase in CFU compared to both an isotype control and untreated conditions (Figure 7C). This suggested that GM-CSF was required for iNKT cell mediated control of Mtb infection in this model. When we added anti-GM-CSF blocking antibodies to co-cultures of WT iNKT cells and Mtb-infected macrophages, we observed a modest decrease in bacterial inhibition by WT iNKT cells in the GM-CSF blockade condition compared to untreated or isotype control, although it was not significant (Figure S5). These results suggest that either the antimicrobial functions of GM-CSF are less crucial in the presence of IFNγ or that there is redundancy in the antimicrobial mechanisms of these two cytokines.

To determine if this iNKT cell effector pathway was also relevant for human cells, we tested the human iNKT cell clone J3N.5. The iNKT cells produced significantly more GM-CSF after co-culture with H37Rv-infected U937 cells, a human monocytic line, than with uninfected U937 cells and this was inhibited by addition of an anti-CD1d blocking antibody (Figure 7D). J3N.5 also produced IFNγ in response to H37Rv-infected U937 cells, and this was similarly inhibited by an anti-CD1d blocking antibody (data not shown). Next, we determined whether iNKT cells produced GM-CSF during aerosol infection in vivo. We isolated cells from the lungs of mice infected with virulent Mtb at serial time points after infection. Pulmonary iNKT cells were identified by TCRβ and CD1d tetramer staining and their production of GM-CSF and IFNγ was assessed by intracellular cytokine staining (ICS) directly ex vivo without further stimulation. The frequency of iNKT cells in the lung did not change greatly over the course of infection, although the total number of iNKT cells increased in parallel with the overall increase in T cell recruitment to the lung (Figure S6A and S6B). Similar to our in vitro observations, CD69 expression increased on iNKT cells in the lung over the course of infection (Figure S6C). A small percentage of iNKT cells in the lungs of naive mice secreted GM-CSF. By two weeks post-infection, there was an increase in both the percentage and absolute number of iNKT cells producing GM-CSF; this was not the case for IFNγ (Figure 7E and 7F). At all time points examined post-infection, a significantly greater percentage of iNKT cells in the lung produced GM-CSF than IFNγ. For example, at week 2, 11.0±1.3% iNKT cells were GM-CSF^+^ compared to 1.0±0.3% IFNγ^+^ iNKT cells (mean ± SEM, p<0.0001). These data demonstrated that iNKT cells are an early source of GM-CSF in the lung during Mtb infection and underscored our in vitro observation that GM-CSF and IFNγ production by iNKT cells are regulated differently during Mtb infection.

In order to evaluate the role of CD1d signaling in the production of GM-CSF by iNKT cells in vivo, we used an adoptive transfer model, in which fluorescently-labeled iNKT cells were injected iv into Mtb-infected WT or CD1d^−/−^ recipients. Downregulation of the iNKT cell TCR usually limits the ability to detect iNKT cells in vivo. This transfer model allowed monitoring of iNKT cells in vivo without the need for CD1d tetramer staining. We detected significantly less GM-CSF and IFNγ production by iNKT cells transferred into Mtb-infected CD1d^−/−^ hosts compared to WT hosts, while CD69 expression was induced on iNKT cells transferred into both WT and CD1d−/− recipients (Figure 7G). Interestingly, we observed a strong CD1d-dependent IFNγ response by the transferred iNKT cells, which we did not detect in the intact mice (Figure 7E and 7F). The transferred iNKT cells were likely to have a stronger response and a lower threshold for activation due to their prior stimulation in vitro. There is recent data, discussed below, that epigenetic modifications at the IFNG locus of iNKT cells may explain why IFNγ is only detected after priming by a strong stimulus [34]. The observation that the in vivo IFNγ response was almost entirely CD1d-dependent suggests that our in vitro infection model may be over-estimating the effect of cytokine-driven stimulation. This experiment also confirmed our in vitro results that activation marker expression and effector functions could be elicited by distinct activating pathways in iNKT cells.

These data showed that both murine and human iNKT cells produced GM-CSF upon recognition of Mtb infection in a CD1d-dependent manner and GM-CSF was a critical component of iNKT cell antimycobacterial function in vitro.

### GM-CSF is sufficient to inhibit Mtb growth

**Figure 8:**
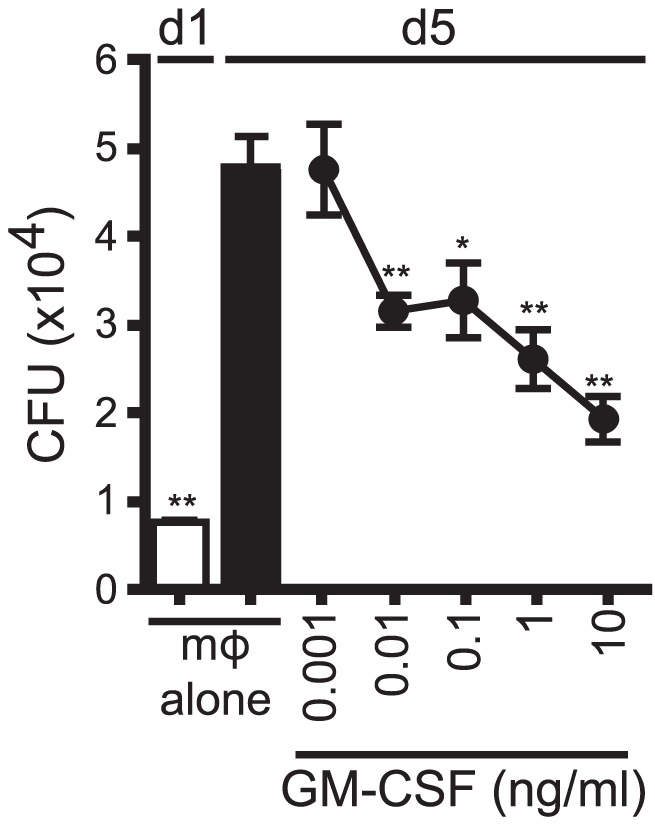
GM-CSF is sufficient for inhibition of Mtb growth. CFU assay for H37Rv-infected murine WT mϕ treated with recombinant GM-CSF from 0.001–10 ng/ml concentration on d1. Error bars indicate mean ± SEM. *P<.05, **P<0.01 (One way ANOVA with Dunnet's post-test, compared to d5 untreated mϕ). Data are representative of four independent experiments.

We next determined whether GM-CSF alone was sufficient to mediate control of Mtb growth. In a dose-dependent manner, recombinant GM-CSF added to infected macrophages was sufficient to inhibit Mtb growth (Figure 8). Recombinant GM-CSF has also previously been reported to inhibit the growth of mycobacterium in human monocyte-derived macrophages (MDM) [35], [36], [37]. The data showed that GM-CSF was sufficient to inhibit bacterial growth in murine macrophages.

## Discussion

We find that iNKT cells cultured with Mtb-infected macrophages inhibit intracellular bacterial growth [18]. This is the only model of which we are aware that allows investigation of the direct antimicrobial effector function of iNKT cells. In the presence of Mtb-infected macrophages, iNKT cells became activated and upregulated the activation markers, CD69 and CD25, and produced IFNγ. While IL-12 and IL-18 produced by infected macrophages induced CD25 and IFNγ expression by iNKT cells, these cytokine signals were not required for iNKT cell control of Mtb. In addition, macrophage expression of CD1d was dispensable for the upregulation of CD25 and only a minor factor in promoting IFNγ production, yet was essential to elicit iNKT cell antimycobacterial activity.

The ability to dissociate iNKT cell activation and IFNγ production from iNKT cell antimicrobial function uncovered a novel antibacterial function of iNKT cells: GM-CSF production. We showed that CD1d-mediated activation was crucial for the production of GM-CSF in response to Mtb infection by both murine and human iNKT cells in vitro, and by murine iNKT cells in vivo. In the absence of IFNγ, GM-CSF was essential for iNKT cell mediated inhibition of Mtb growth, and GM-CSF alone was sufficient for bacterial control in vitro. Interestingly, under conditions where either CD1d signaling or GM-CSF was blocked during iNKT cell co-culture there was still on average a 30% reduction in CFU. In the case of antibody blocking, inefficient inhibition during the five day assay may be a technical confounder. In addition, this data points to two alternative biological explanations. First, signals apart from TCR activation may contribute to the production of GM-CSF by iNKT cells (Figure 7B). Cytokines such as IL-12 plus IL-18 stimulate iNKT cells to produce GM-CSF (data not shown). Second, there may be molecules other than GM-CSF and IFNγ that are produced by iNKT cells and activate infected macrophages to inhibit bacterial growth. While GM-CSF is unlikely to be the only iNKT cell effector function that inhibits Mtb replication, our experiments demonstrate that it is a dominant antimicrobial pathway during Mtb infection.

Although signaling by IFNγ is crucial for control of Mtb, clinical data shows that IFNγ present at the site of ongoing infection is inadequate to clear bacteria and IFNγ levels produced by CD4^+^ T cells do not correlate with disease progression or protection provided by BCG vaccination [38], [39], [40]. An implication of these studies is that alternative pathways exist that control Mtb. A number of studies have demonstrated IFNγ-independent mechanisms of control by CD4^+^ and CD8^+^ T cells [41], [42], [43]. Using iNKT cells as a model, we evaluated IFNγ-independent pathways of control in the innate immune compartment.

Two previous studies find GM-CSF to be required for host resistance in vivo based on the greater susceptibility of GM-CSF^−/−^ mice compared to WT mice [44], [45]. Although alveolar macrophages and type II epithelial cells were presumed to be the dominant source of GM-CSF in the lung, ectopic expression of GM-CSF driven by the surfactant C promoter did not fully rescue the susceptibility of GM-CSF^−/−^ mice, suggesting that GM-CSF from other sources might be important for immunity. T cells are an important source of GM-CSF, and in addition to iNKT cells, other innate-like T cells produce GM-CSF during Mtb infection (manuscript in preparation). We have also found that conventional CD4^+^ T cells in the lungs of Mtb infected mice produce GM-CSF and may replace innate lymphocytes as a source of GM-CSF as the immune response to Mtb evolves. This may be one explanation for why iNKT cells are redundant during Mtb infection. Although GM-CSF restricts bacterial replication in human macrophages, its role as an effector molecule during clinical infection is harder to discern. Importantly, recent clinical data indicates that the development of anti-GM-CSF neutralizing antibodies are a form of acquired immunodeficiency associated with cryptococcal meningitis and pulmonary tuberculosis in otherwise normal individuals [46]. This suggests that inhibition of GM-CSF signaling may increase clinical susceptibility to Mtb and other pulmonary pathogens.

To put our in vitro experiments in context, cytokines induced by Mtb-infected macrophages, which include IL-12 and IL-18, are drivers of IFNγ and other cytokine production by iNKT cells, independent of TCR activation (e.g., in an antigen-independent manner). An important question is whether this mechanism is relevant in vivo. In vitro, our assays use numerous macrophages that are uniformly infected, with the consequence that the effective cytokine concentration that the iNKT cells are exposed to could be higher than is relevant in vivo. Furthermore, the iNKT cell lines are primed to produce IFNγ because they have been repeatedly stimulated in vitro with αGalCer (see below). Interestingly, the iNKT cell lines produced both IFNγ and GM-CSF after short-term adoptive transfer into Mtb-infected mice, but only in a CD1d-dependent manner. Cytokine production was only observed when the iNKT cells were transferred into Mtb-infected WT mice but not when transferred into infected CD1d^−/−^ mice. Thus, although CD1d-independent GM-CSF and IFNγ was observed in vitro, TCR signaling is crucial in vivo during infection to stimulate iNKT cells to produce cytokines.

After a strong stimulus such as αGalCer, human iNKT cell clones produce both GM-CSF and IFNγ; in contrast, GM-CSF dominates after a weak or autoreactive stimulus [47]. GM-CSF production by iNKT cells plays an important role in the maturation of DC, which has been linked to effective T cell priming [47], [48]. Wang et al found that in resting human iNKT cells the *CSF2* (GM-CSF) locus already had high histone H4 acetylation, indicative of chromatin availability, while the *IFNG* (IFNγ) locus had low histone H4 acetylation, which was increased only after strong stimulation [34]. In contrast to the iNKT cell lines, we observed that endogenous polyclonal iNKT cells in the lungs of Mtb-infected mice more frequently produced GM-CSF than IFNγ, consistent with the idea that iNKT cells are poised to produce GM-CSF more readily than IFNγ, particularly when exposed to weak TCR agonists. This study further confirms that different iNKT cell effector functions may require different stimuli and that epigenetic modifications may explain at least part of this phenomenon.

It is clear that there is great heterogeneity in iNKT cell responses and that part of this variability comes from the diversity in activating stimuli iNKT cells encounter. We observed two distinct modes of activation that initiated different effector functions: (1) the IL-12/IL-18 pathway activated iNKT cells to produce IFNγ, and (2) a TCR-dependent pathway elicited antimycobacterial activity. Interestingly, in the context of Mtb infection, the conventional markers of iNKT cell activation did not correlate with effector function and our data indicate that GM-CSF production may be a better marker of TCR-dependent iNKT cell effector function than IFNγ. Using transcriptional profiling, Cohen et al elegantly showed that iNKT cells share features of both NK cells and T cells [49]. Here we show that during the host response to infection, different stimuli may trigger different iNKT cell effector programs characteristic of NK cells (e.g. activation by IL-12 and IL-18) or T cells (e.g. TCR activation). Future insight into iNKT cell immunity will require better understanding of how different activation stimuli can dictate subsequent effector functions and may facilitate the discovery of other novel antimicrobial roles for iNKT cells.

## Materials and Methods

### Ethics statement

All mice were bred and maintained using standard humane animal husbandry protocols. All animal experiments were performed in accordance with relevant guidelines for the care and handling of laboratory animals and were approved by the Dana Farber Cancer Institute Animal Care and Use Committee (Animal Welfare Assurance number A3023-01) under Public Health Service assurance of the Office of Laboratory Animal Welfare guidelines. Human blood collected from healthy donors was purchased from Research Blood Components (Boston, MA), and its use was approved by the Institutional Review Board of Brigham and Women's Hospital (Human Subjects Assurance FWA00000484). Written informed consent was obtained for each donor by Research Blood Components.

### Mice

C57Bl/6 WT, IFNγ^−/−^, IFNγR^−/−^, TNFR1/2^−/−^, Perforin^−/−^, and Fas^−/−^ mice were obtained from Jackson Laboratories. Vα14-Jα281 transgenic mice were provided by Dr. Albert Bendelac [50]. CD1d^−/−^ mice were provided by Dr. Mark Exley [51]. MyD88^−/−^ mice were provided by Dr. Koichi Kobayashi [52].

### Macrophage isolation and culture

Thioglycollate (TGL)-elicited peritoneal macrophages were lavaged 4–5 days after 3% intraperitoneal TGL injection and then isolated by positive selection with CD11b microbeads and magnetic columns (Miltenyi Biotec). Purified cells were over 95% F4/80^+^ CD11b^+^, as determined by flow cytometry. Bone marrow-derived macrophages (BMDM) were differentiated from bone marrow after 7 days in RPMI supplemented with 20% L929 cell supernatant. U937 cell line was grown in complete media. Macrophages were seeded at 5×10^5^ cells/well in 24-well culture plates or 1×10^5^ in 96-well culture plates in complete RPMI 1640 medium (Invitrogen Life Technologies) supplemented with 10% fetal calf serum (HyClone). For transwell assays, macrophages were seeded at 2×10^5^ in 0.4 µm cell culture inserts for 24-well plates (BD Bioscience).

### iNKT cells and HMNC

iNKT cell lines were derived as previously reported [53]. T cells were selected from splenocytes using the Pan T Isolation Kit (Miltenyi Biotec) and then cultured overnight at 37°C. The next day, T cells were labeled with PE-conjugated CD1d tetramer loaded with PBS-57 lipid antigen (National Institutes of Health Tetramer Core Facility) and sorted using anti-PE beads (Miltenyi Biotec). The purity of iNKT cells was higher than 95%. iNKT cells were then cultured with irradiated αGalCer-pulsed BMDCs in 24-well plates in complete RPMI medium with 10% FBS. Three to four days later, 1 ng/ml IL-2 (R&D Systems) and 10 ng/ml IL-7 (PeproTech) were added. iNKT cells were rested for at least 18 days before use. Human iNKT cell clones were derived and cultured as previously described [7]. Hepatic mononuclear cells (HMNCs) were isolated from mouse liver perfused with PBS and homogenized through 70 υm cell strainer to single cell suspension. After centrifugation, the cells were resuspended in 30% Percoll and overlayed onto 80% Percoll layer (Sigma). The interface containing the lymphocytes was collected and washed before use.

### Mtb in vitro culture and infection

H37Rv was grown and prepared as previously described [18]. Bacteria was counted and added to macrophages at an effective multiplicity of infection (MOI) of 0.2 for CFU experiments (or higher for ELISA and FACS assays) for two hours. Cultures were washed three times to remove extracellular bacteria. Infected macrophages were cultured overnight and iNKT cells or other conditions were added on d1. For CFU measurement, cells were lysed with 1% Triton X-100/PBS and lysate from quadruplicate conditions were plated in serial dilutions on Middlebrook 7H10 agar plates (ThermoFisher Scientific), and cultured at 37°C for 21 days. Infected macrophages were treated with the following reagents: caspase-3 inhibitor and caspase inhibitor negative control (Calbiochem), anti-mouse-IL-12p40 (C17.8; Biolegend), anti-mouse-IL-18 (93-10C; MBL), anti-mouse-CD1d (19G112.2) [54], anti-human-CD1d (CD1d42; BD Pharmingen), anti-mouse-GM-CSF (MP1-22E9; Biolegend), recombinant murine GM-CSF (Peprotech), and IFNγ (murine, Peprotech; human, Biolegend). αGalCer was kindly provided by Gurdyal S. Besra.

### % CFU reduction

To compare inhibition of bacterial growth across multiple experiments, % CFU reduction was calculated. 100% CFU reduction on d5 indicates complete inhibition of bacterial growth to d1 levels while 0% CFU reduction indicates no change in bacterial growth from untreated macrophages. *% CFU reduction = 100×[CFU_(untreated mf-d5)_−CFU_(treated mf-d5)_]/[(CFU_(untreated mf-d5)_−CFU_(untreated mf-d1)_]*

### In vivo aerosol infections

In vivo infections were performed using virulent Mtb (Erdman strain). Mice infected with Mtb were housed under BSL3 conditions. For each infection, a bacterial aliquot was thawed, sonicated twice for 10 s, and then diluted in 0.9% NaCl/0.02% Tween 80. A 15-ml suspension of *M. tuberculosis* was loaded into a nebulizer (MiniHEART nebulizer; Vortran Medical Technology) and mice were infected via the aerosol route with a nose-only exposure unit (Intox Products) and received ∼50–100 CFU/mouse. Mice were euthanized by CO_2_ inhalation and lungs were aseptically removed after perfusion of 10 ml of sterile RPMI into the right ventricle of the heart. Lung mononuclear cells were obtained by mechanical disruption using a gentleMACS dissociator (Miltenyi Biotec) followed by incubation in collagenase (Sigma-Aldrich) for 30 mins at 37°C. Cells were isolated by forcing suspensions through a 70 µM cell strainer and then enumerated in 4% trypan blue with a hemacytometer. Samples used for ICS were incubated for 4 hours at 37°C with IL-2 and Brefeldin A (GolgiPlug, BD Biosciences).

### Adoptive transfer of iNKT cell lines

3–5×10^6^ iNKT cells were stained with Cell Proliferation Dye eFluor 450 (eBioscience) following the manufacturer's protocol and then iv injected via tail vein into Mtb-infected WT or CD1d^−/−^ mice. Twenty hours later, mice were euthanized and lungs were removed and digested. Lung mononuclear cells were stained by surface and ICS protocols and paraformaldehyde fixed. Untransferred iNKT cells cultured in standard media were similarly treated to ascertain baseline activation. Flow cytometry gating strategies allowed for separation of endogenous versus transferred iNKT cells independent of tetramer staining by the presence of fluorescent dye.

### Flow cytometry and ICS

Cells were first incubated with CD16/CD32 (FcBlock; BD Biosciences). Surface staining for in vitro experiments included antibodies for mouse CD69 (H1.2F3), CD25 (PC61), F4/80 (BM8), CD1d (1B1), and isotype controls (all from Biolegend). Surface staining of lung mononuclear cells included antibodies for mouse TCRβ (H57-597), CD69, CD3 (17A2), CD19 (6D5) and isotype controls (all from Biolegend). PBS-57-loaded and control PE- and APC-conjugated CD1d tetramers were provided by the National Institute of Allergy and Infectious Diseases Tetramer Facility (Emory University Vaccine Center). After tetramer staining, ICS with antibodies specific for mouse GM-CSF (MP1-22E9; eBioscience) and IFNγ (XMG1.2; Biolegend) was performed following fixation with 4% paraformaldehyde and permeabilization with Perm/Wash buffer (BD Biosciences). Data were collected using FACSCanto (BD Biosciences) and analyzed with FlowJo (Tree Star, Inc.).

### ELISA, nitric oxide, and Bioplex immunoassays

Culture supernatants were filtered through 0.2 µm filter to remove any bacteria. IFNγ, IL-12p40, and GM-CSF ELISAs were done in accordance with the manufacturer's instructions (Biolegend), and absorbance was recorded at 450 nm on SoftMax Pro ELISA analysis software (Molecular Devices). Nitric oxide (NO) production was measured using the Griess reaction to detect nitrite, a stable breakdown product of NO, as described previously [18]. For size fractionation, Amicon Ultra-15 Centrifugal Filter Units with 10 kDa and 50 kDa cutoff were used (EMD Millipore). Bioplex immunoassay was done in accordance with the manufacturer's instructions (BioRad).

### Statistical analysis

Data was analyzed by one-way ANOVA (95% confidence interval) with Dunnett's post-test (for comparison against a single control) or Tukey post-test (for comparison between all conditions) or unpaired Student's t-test. Analysis was performed using GraphPad Prism software.

## Supporting Information

Figure S1 **iNKT cell activation after Mtb infection is observed with primary iNKT cells and bone marrow derived-macrophages (BMDM).** (A) HMNC were cultured either alone, with uninfected thioglycollate-elicited peritoneal mϕ (TGL-PM), or H37Rv-infected TGL-PM for 24 hours and gated on CD1d-tetramer^+^CD3^+^ population. Fold change in CD69 MFI measured over HMNC cultured alone. Supernatant was harvested at 24 hours and IFNγ measured by ELISA. (B) iNKT cells were cultured with uninfected or H37Rv-infected WT bone marrow derived macrophages (BMDM) for 24 hours. Fold change in CD69 MFI over iNKT cells cultured alone. Supernatant was harvested at 24 hours and IFNγ measured by ELISA. Error bars indicate mean ± SEM. The data is from a single experiment. Note that the IFNγ release is less than in Figure 1. This difference is likely to arise from the use of primary HMNCs in Figure S1A and different APCs in Figure S1B. Figure S1A uses hepatic mononuclear cells (HMNC), as a source of primary uncultured iNKT cells, to demonstrate that the culture conditions of the primary iNKT cell lines did not alter the behavior of the cells. iNKT cells are a small fraction of conventional T cells in most sites of the body. In the liver, iNKT cells make up about 10–20% of the total lymphocytes; therefore, the difference in the magnitude of the IFNγ response may be due to the use of a lower number of iNKT cells. Secondly, the use of cultured iNKT cells (in Fig. 1) may generate a stronger response because they act as if ‘primed’ because they are repeatedly stimulated in the presence of cytokines. Fig. S1B used BMDM to show that Mtb-infected APCs other than TGL-PM stimulate iNKT cells. BMDM are not as activated as TGL-PM, which contributes to the difference in the level of responses in this experiment.
(EPS)

Figure S2 **WT and IFNγ^−/−^ iNKT cells both inhibit Mtb growth in vitro at comparable levels.** Compiled data from CFU assays d5 and d7 post-infection for H37Rv-infected WT mϕ with WT or IFNγ^−/−^ iNKT cells added on d1. The CFU reduction for WT and IFNγ^−/−^ iNKT cells on d5 was 60.0±4.7%, and 60.1±4.0%, respectively. On d7, the CFU reduction was 81.9±5.4%, and 67.9±6.2%, respectively. Error bars indicate mean ± SEM. (Unpaired Student's t-test, p = NS). The data are compiled from 13 (d5) and four (d7) independent experiments.
(EPS)

Figure S3 **Conditioned media fractions from α-GalCer stimulated IFNγ^−/−^ iNKT cells inhibit bacterial growth.** (A) Size fractionation strategy for conditioned media samples using 50 kDa and 10 kDa Amicon Ultra-15 Centrifugal Filter Units. (B) CFU assay for H37Rv-infected WT mϕ treated on d1 with whole and size fractionated conditioned media samples from IFNγ^−/−^ iNKT cells stimulated for 24 hours with untreated or αGalCer-loaded WT or CD1d^−/−^ mϕ at 1∶50 dilution. (C) Cytokines measured in whole and size fractionated conditioned media samples from IFNγ^−/−^ iNKT cells stimulated for 24 hours with untreated or αGalCer-loaded WT or CD1d^−/−^ mϕ. Cytokines were measured using Bioplex immunoassay. Error bars indicate mean ± SEM. *P<0.05, **P<0.01 (One-way ANOVA with Dunnet's post-test, compared to d5 untreated mϕ). The data are representative of two independent experiments.
(EPS)

Figure S4 **WT iNKT cells produce GM-CSF following Mtb infection.** Murine GM-CSF measured in supernatants after 24-hr co-culture of WT iNKT cell line with uninfected and H37Rv-infected WT mϕ. ***P<0.001 (One-way ANOVA with Dunnet's post-test, compared to uninfected mϕ). The data are representative of three independent experiments.
(EPS)

Figure S5 **Control of intracellular Mtb replication by WT iNKT cells in the presence of anti-GM-CSF blocking antibody.** WT iNKT cells were added to H37Rv-infected WT mϕ on d1 without additives, or in the presence of anti-GM-CSF blocking antibody or nonspecific isotype control antibody (25 µg/ml). CFU were determined on d5. The data are compiled from three independent experiments with four replicates per condition, and normalized to calculate the percent CFU reduction. Statistical analysis was performed using a one-way ANOVA and was not significant. Error bars indicate mean +/− SEM.
(EPS)

Figure S6 **iNKT cells are found in the lung after aerosol Mtb infection.** Lung mononuclear cells from WT Mtb-infected mice were stained and fixed. iNKT cells were identified as TCRβ^+^ and CD1d-tetramer^+^. Number (A) and percentage (B) of iNKT cells in the lung were measured. (C) CD69 MFI measured on iNKT cell subset. The data are representative of two independent experiments with 5 mice each.
(EPS)
